# Anti-Cancer Effects of *Panax ginseng* Berry Polysaccharides *via* Activation of Immune-Related Cells

**DOI:** 10.3389/fphar.2019.01411

**Published:** 2019-11-26

**Authors:** Dae-Young Lee, Chan Woong Park, Sue Jung Lee, Hye-Ryung Park, Su Hwan Kim, Seung-U Son, Jiyong Park, Kwang-Soon Shin

**Affiliations:** ^1^Department of Food Science and Biotechnology, Kyonggi University, Suwon, South Korea; ^2^R&D Center, Vital Beautie Research Institute, AmorePacific Corporation, Yongin, South Korea; ^3^Department of Biotechnology, Yonsei University, Seoul, South Korea

**Keywords:** polysaccharide, ginseng berry, immunostimulation, metastasis, macrophage, NK cell

## Abstract

*Panax ginseng* has long been used as natural medicine and health food all over the world. Cancer is a major cause of death worldwide and its prognosis likely depends on the immune system during tumor treatment. In this study, ginseng berry polysaccharides were evaluated for their immunostimulant and anti-cancer effects. Ginseng berry polysaccharide portion (GBPP) was used to investigate its effects on anti-complementary activity, peritoneal macrophage activation, and natural killer (NK) cell cytotoxicity. Moreover, both intravenous (*i.v.*) and oral administration of GBPP prior to B16-BL6 melanoma implantation in mice was evaluated. GBPP significantly increased the anti-complementary activity and cytokine production including interleukin (IL)-6, IL-12, and tumor necrosis factor (TNF)-α, dose-dependently. Splenocytes obtained after *i.v.* administration of GBPP showed cytolytic activity in Yac-1 cells in proportion to the E/T ratio. In addition, GBPP enhanced the production of interferon (IFN)-γ and granzyme B of NK cells. For the experimental lung cancer, compared with control mice, GBPP delivered by *i.v.* suppressed cancer by 48% at 100 μg/mouse, while a 37% reduction was achieved by oral administration. Deficient of NK cells in animal model demonstrated that the anti-cancer effect of GBPP was through NK cell activation. Results of this study suggest that ginseng berry polysaccharides, owing to their modulation of the immune response, can be a potential curative applicant for the prevention and treatment of tumors.

## Introduction

*Panax ginseng* is widely used around the world because of its pharmacological effects on the immune system, diabetes, blood circulation, atherosclerosis, and sexual activity (Nam, 2002). Ginseng contains medicinal components such as saponins, polysaccharides, polyacetylenes, phenols, gominins, acidic peptides, and carbohydrates (Wu and Zhong, 1999). Each part of the ginseng plant has a unique ginsenoside profile, which means that the various parts likely exert different pharmacological effects (Attele et al., 1999). Recently, some studies have shown that the berry of *Panax ginseng* has a much stronger pharmacological activity than its root. Dey et al. investigated that, compared to ginseng root, ginseng berry exhibits more potent anti-hyperglycemic activity, and only ginseng berry shows significant anti-obesity effects in *ob/ob* mice (Dey et al., 2003). Studies on ginseng berries have investigated their antiaging action, therapeutic effects on gastric ulcers, immunological effects on lupus erythematosus, and anti-stress effect of the saponins found in them (Huo, 1984; Zhang et al., 1984; Yang and Zhang, 1986; Zhao et al., 1991). In addition, ginseng berry has various pharmacological properties such as heart protection, vasodilation, anticoagulation, anti-stress activity, and neuroprotection. In a series of biochemical investigations, several major components, especially various ginsenosides, were isolated from ginseng berry (Wang et al., 2004). The anti-diabetic and anti-obesity effects of Asian ginseng berry have been demonstrated in diabetic and obese ob/ob mice, and the observed effect was attributed to ginsenosides. However, the pharmacological properties of ginseng berry have not been as extensively investigated as those of ginseng root (Attele et al., 2002; Wang et al., 2007). There is strong evidence to suggest that ingesting phytochemicals that are naturally present in fruits or vegetables is more effective than purified products or extracts of the same chemicals (Boivin et al., 2009). In addition, water-extractable polysaccharides can also contribute to the beneficial health effects of fruits such as ginseng berry (Ross et al., 2015). There is already evidence for chemoprevention and anti-cancer effects of numerous polysaccharides extracted from various herbs, which are exerted *via* immune response of host organism (Liu et al., 2011; Bai et al., 2012; Li et al., 2013). Purified ginseng berry polysaccharide extract has been examined in C57BL/6 mice model to investigate its anti-tumor activity and immune regulation (Wang et al., 2015). Studies on the anti-hyperglycemic properties of ginseng berry polysaccharides have also been carried out (Xie et al., 2004). However, very few studies have investigated the physiological activities of ginseng berry polysaccharides, compared with those on ginseng root polysaccharides. Wan et al. evaluated the activities of polysaccharides of ginseng berry on plasma lipid levels, chemically induced intestinal inflammation, and neoplasm and cancer chemoprevention in multiple experimental models (Wan et al., 2017).

In this study, the anti-cancer effects of ginseng berry polysaccharides were investigated that are likely exerted *via* regulation of immune-stimulating properties. As the test material, we used ginseng berry polysaccharide portion (GBPP), wherein molecules with a molecular weight < 20 kDa were removed.

## Materials and Methods

### Material and Animals

Berries of *Panax ginseng* C.A. Mey. were collected from Keumsan, Chungnam Province, Korea (2015). The YAC-1 cell line was obtained from the Korean Cell Line Bank (Seoul, Korea). Six-week-old female BALB/c mice were purchased from Saeronbio Inc. in Korea and kept under pathogen-free conditions. The mice were group-housed (5–6 per cage) under a reversed light-dark cycle. The room temperature was 20–25°C and the humidity was 30 ± 5%. The mice were given free access to laboratory diet (Saeronbio Inc.) and water. The mice maintained and studied according to protocols approved by the Committee on the Ethics of Animal Experiments of Kyonggi University (2016-002 and 2017-005) and adhere to Guide for the Care and Use of Laboratory Animals (NIH Publication Nos. 85–23, 1996).

### Preparation of Ginseng Berry Polysaccharide Portion

**Figure 1:**
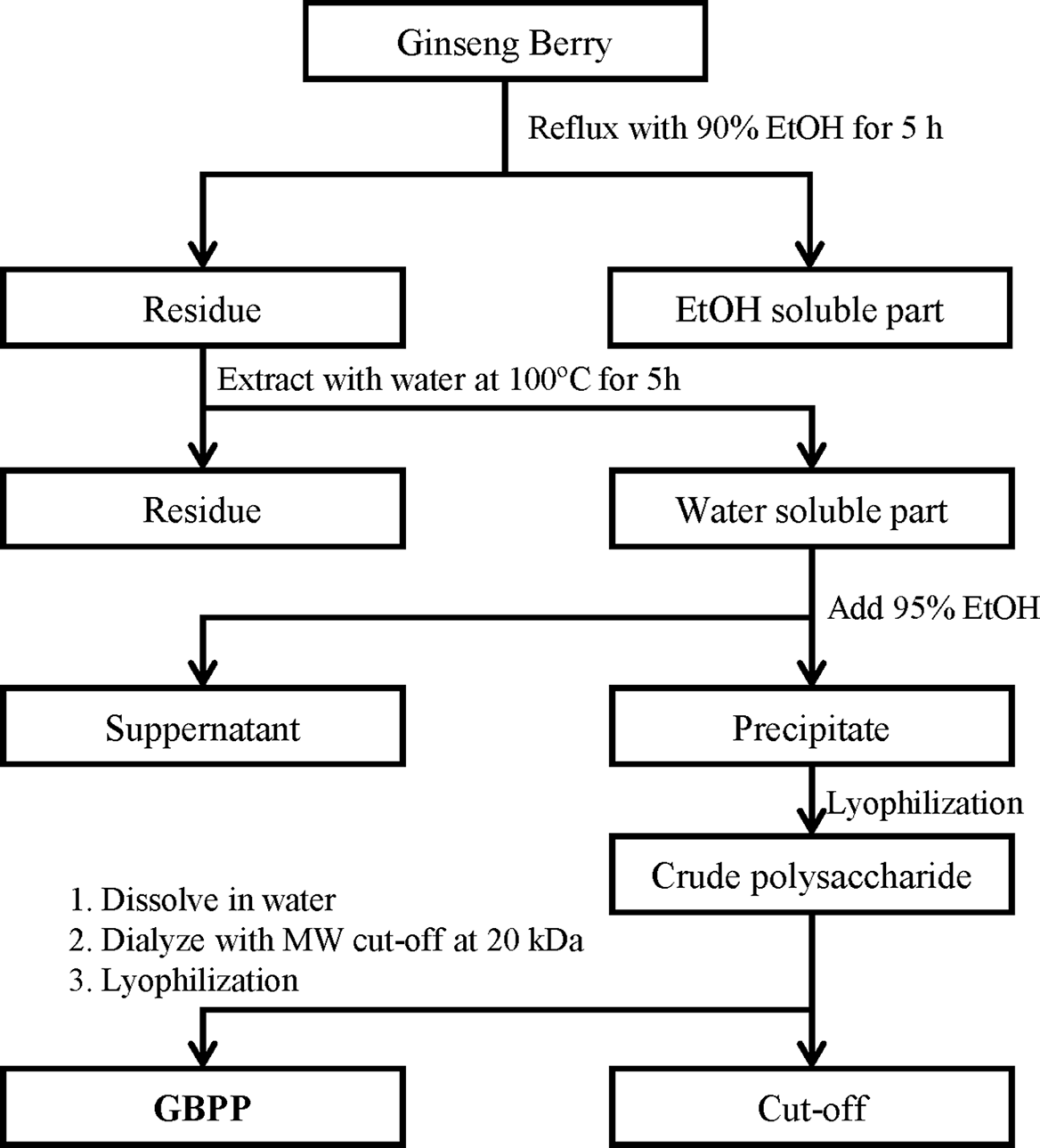
Schematic diagram of the preparation of ginseng berry polysaccharide portion (GBPP) from *Panax ginseng* berry.

The GBPP was obtained from Amore Pacific Corp. (Seoul, South Korea). A voucher specimen was deposited at the InfoBoss Cyber Herbarium (IN, Seoul, South Korea) and the voucher number is IBS-00011. Briefly, ginseng berry was refluxed using 90% ethanol for 5 h. The residue was then treated with water at 100°C for 5 h. By adding four volumes of 95% ethanol to the supernatant, the polysaccharides were precipitated and dried to obtain crude ginseng berry polysaccharide extract. The crude polysaccharide extract was dissolved in water and dialyzed with a membrane (molecular weight cut-off; 20 kDa, Spectrum Laboratories Inc., Rancho Dominguez, CA, USA). The dried powder (GBPP) was collected after lyophilization and stored at −20°C until use (**Figure 1**).

### General Analysis

Total carbohydrate concentration was determined by the phenol-sulfuric acid method (DuBois et al., 1956), with galactose as a reference. The uronic acid content was measured following the *m*-hydroxybiphenyl method (Blumenkrantz and Asboe-Hansen, 1973), using galacturonic acid as a reference. The 2-*keto*-3-deoxy-D-manno-octulosonic acid (KDO) content was determined following a modified thiobarbituric acid (TBA) method (Karkhanis et al., 1978), using KDO as a reference. The protein content was analyzed following the Bradford method (Bradford, 1976), using bovine serum albumin as a reference. The monosaccharide composition of the polysaccharides was analyzed following the partially modified alditol acetate method of Jones and Albersheim (Jones and Albersheim, 1972), using gas chromatography (GC) equipment (6000 series, Young-Lin Co., Anyang, Korea) equipped with an SP-2380 capillary column (0.2 µm × 0.25 mm × 30 m; Supelco, Bellefonte, PA, USA) and a flame ionization detector (FID). The temperature program of the GC was: 60°C for 1 min, 60 → 220°C (30°C/min), 220°C for 12 min, 220 → 250°C (8°C/min), and 250°C for 15 min. The molar ratio of monosaccharides was calculated from peak areas and response factors.

The content of arabino-β-D-3,6-galactan in the polysaccharide portion was analyzed using β-D-glycosyl Yariv reagent according to the procedure described by Holst and Clarke (Holst and Clarke, 1985).

### Measurement of Anti-Complementary Activity

Complement activation was measured following the method of Mayer with some modifications (Mayer, 1964; Park et al., 2013). Normal human sera (NHS) were prepared freshly using samples from healthy adult volunteers from Kyonggi University, Korea. Freshly drawn whole blood was treated following centrifugation at 800 ×*g* for 10 min after allowing it to stand for 30 min. The supernatant NHS was pooled, divided into 1-ml aliquots, and preserved at −70°C before use for assays. Frozen preparations were thawed immediately before use. Gelatin-Veronal buffered saline (GVB^2+^) (pH 7.4), containing 500 µΜ Mg^2+^ and 150 µM Ca^2+^, were prepared. Polysaccharides (50 µl) in ice water were incubated with NHS (50 µl) and GVB^2+^ (50 µl). Mixtures were incubated at 37°C for 30 min, and 350 µl of GVB^2+^ was added to them. Immunoglobulin M-hemolysin-sensitized sheep erythrocytes (EA) cells, adjusted to 1×10^8^ cells/ml (250 µl), were added to the mixtures with serial dilution (10–160 fold), followed by incubation at 37°C for 60 min. Afterwards, 2.5 ml of phosphate buffered saline (PBS, pH 7.4) was added and centrifugation at 700 ×*g* was carried out for 10 min. The absorbance of supernatants was detected at 412 nm. NHS was separately incubated with both water and GVB^2+^ to provide a control. The anti-complementary activity of the polysaccharide was expressed as a percentage inhibition of 50% of total complement hemolysis (ITCH_50_) of the control:

$${\mathrm{ITCH}}_{50}(\%)\;=\;\left[\left({\mathrm{TCH}}_{50}of\;\mathrm{control}\;-\;{\mathrm{TCH}}_{50}\mathrm{treated}\;\mathrm{with}\;\mathrm{sample}\right)/{\mathrm{TCH}}_{50}of\;\mathrm{control}\right]\times 100$$

### Peritoneal Macrophage Activation

#### Isolation of Macrophages

Peritoneal macrophages of BALB/c mice were isolated as represented previously (Shin et al., 2017). The isolated cells were incubated in Roswell Park Memorial Institute (RPMI)-1640 medium with 10% heat-inactivated fetal bovine serum (FBS) and seeded at a density of 2 × 10^5^ cells/well for 2 h in a 5% humidified CO_2_ incubator. The adherent cells were then washed with phosphate buffered saline (PBS) and pretreated with various concentrations of GBPP for 24 h.

#### Cytotoxicity Toward Macrophages

Cytotoxicity was assessed using the Cell Counting Kit-8 (CCK-8) (Dojindo Molecular Technologies, Gaithersburg, MD, USA). CCK-8 was added to each well, followed by incubation at 37°C for 30–60 min in a CO_2_ incubator. The absorbance was measured at 450 nm using a microplate reader (Molecular Devices Co., CA, USA).

#### Cytokine Analysis

Peritoneal macrophages were seeded at a density of 2 × 10^5^ cells/well in 48-well plates and treated with various concentrations of GBPP for 18 h. A positive control was lipopolysaccharide (LPS). The supernatant was collected and then centrifuged. The final supernatant was used for cytokine analysis. Production of interleukin (IL)-6, IL-12, and tumor necrosis factor (TNF)-α was determined using sandwich enzyme-linked immunosorbent assay (ELISA) sets (Becton-Dickinson and Co., Franklin Lakes, NJ, USA), according to the manufacturer’s protocols.

#### Splenic Natural Killer-Cell Activity

To analyze the effect of GBPP on natural killer (NK)-cell activation in mouse splenic cells, GBPP was intravenously administered to BALB/c mice. Before the treatment, GBPP was filtered through 0.22 µm cellulose acetate syringe filters (Advantec, Tokyo, Japan). Splenic cells from mice were co-cultured with YAC-1 cells (1×10^5^ cell/ml) to obtain E/T ratios of 100:1, 50:1, and 25:1 (1×10^7^ cell, 5×10^6^ cell, and 2.5×10^6^ cell/ml) in 96-well plates. The E/T ratio indicates the ratio of effector cells (splenic cells) to target cells (YAC-1 cells). The supernatant was collected following centrifugation (900 ×g, 5 min) and combined with lactate dehydrogenase (LDH) reagent (Promega Co., Madison, WI, USA) in a new 96-well plate. After incubation, the plate was analyzed at 490 nm using a microplate reader. The percentage of NK cell cytotoxicity was determined using the following formula: cytotoxicity (%) = [(absorbance value of experimental group − absorbance value of control group)/(absorbance value of untreated group − absorbance value of control group)] × 100. The expression of interferon (IFN)-γ and granzyme B was assessed using ELISA kits (BD Biosciences, San Diego, CA, USA), according to the manufacturer’s protocols.

### Anti-Cancer Effect

#### Experimental Lung Cancer

The inhibition activities of GBPP on lung cancer were assessed after the mice were treated with GBPP both orally (*p.o.*) (daily for 15 days) and intravenously (*i.v.*) (twice, 2 days prior to or 1 day after *i.v.* tumor inoculation). Tentative lung cancer was estimated based on *i.v.* inoculation with B16-BL6 melanoma cells (2.7 × 10^4^ cells per mouse) into syngeneic BALB/mice, followed by sacrifice at 14 days after tumor dissemination. The lung samples were fixed in Bouin’s solution (Choon et al., 1994) and the tumor colonies were counted under a dissecting microscope.

#### Depletion of Natural Killer Cells ***In Vivo***

NK cell depletion in animal model was carried out in response to a previously described method (Kasai et al., 1981). Mice were intraperitoneally injected with 500 µl/mouse of rabbit anti-asialo GM1 serum (Wako Pure Chemicals Industries, Ltd., Japan) 2 days before tumor inoculation. The protocol after the anti-asialo treatment was the same as that for the tentative lung cancer test.

#### Cytotoxic T Lymphocyte Activity

After NK cell depletion assay using anti-asialo GM1 serum, the mice were sacrificed 14 days after tumor inoculation, and their spleen enucleated. Thereafter, splenocytes suspensions were added to the Colon26-M3.1 cells (1 × 10^4^ cells/well) to obtain effector cell-to-target cell (E/T) ratios of 100:1, 50:1, and 25:1 in round-bottomed 96-well plates, and co-cultured for 6 h. After centrifugation, the culture supernatants were mixed with LDH solution and absorbance value of each well was recorded at 490 nm. The percentage of CTL cytotoxicity was calculated using the following formula:

CTL cell cytotoxic activity (%) = [(experimental group release − effector cell spontaneous release − target cell spontaneous release)/(target cell maximum release − target cell spontaneous release)] × 100.

### Data Analysis

Data are expressed as mean ± standard deviation (SD). Differences among groups were evaluated using a one-way analysis of variance (ANOVA), followed by Duncan’s multiple range test. In all instances, values of p < 0.05 were considered significant.

## Results

### Chemical Composition of Ginseng Berry Polysaccharide Portion

**Figure 2:**
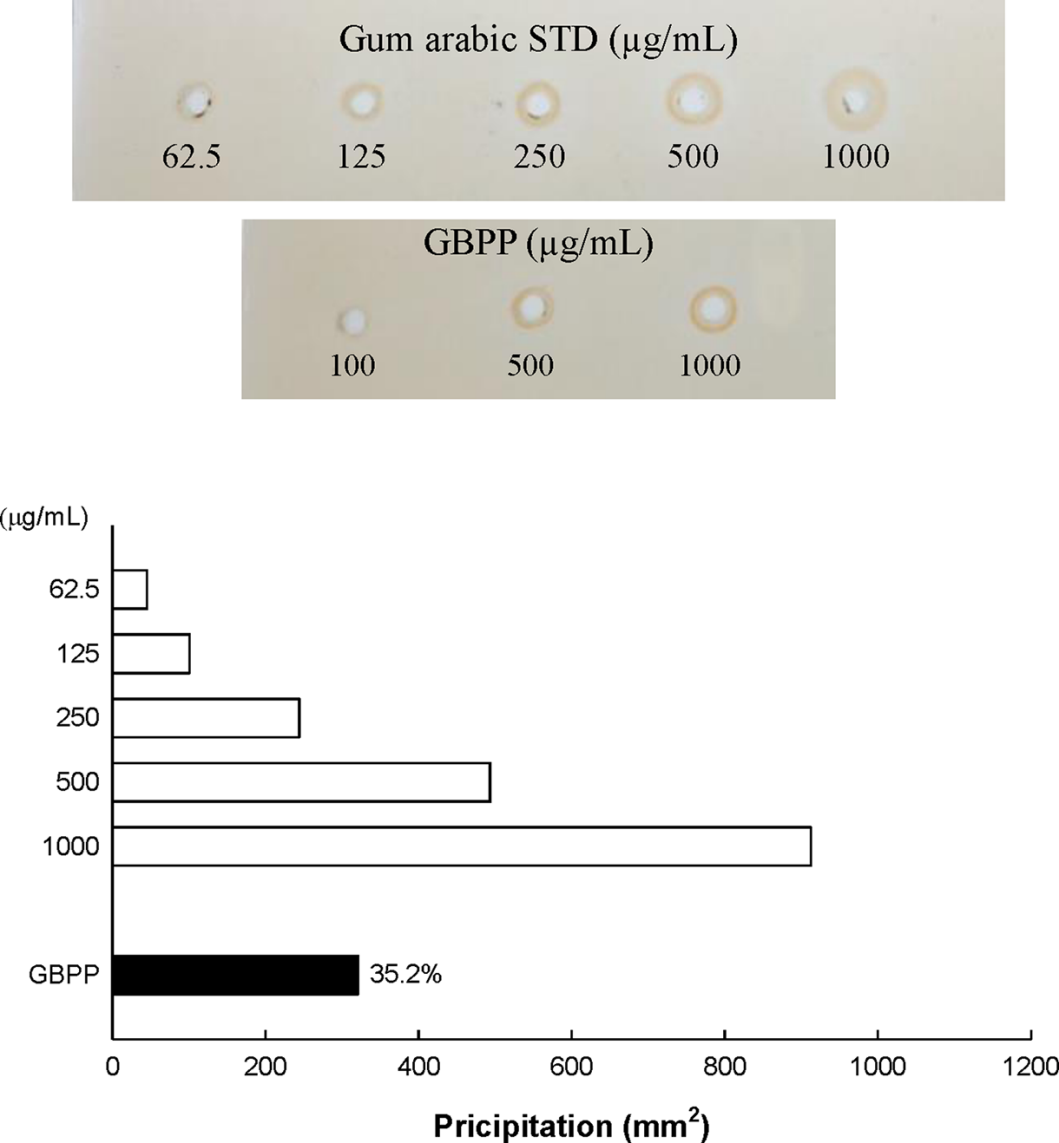
Content of arabino-β-D-3,6-galactan (type II arabinogalactan; AG-II) in ginseng berry polysaccharide portion, assessed using single radial gel diffusion and the β-D-glucosyl Yariv reagent.

Water extract (ginseng berry crude polysaccharide; GBCP) containing high-molecular weight (MW) polysaccharides and a few low MW substances were isolated from ginseng berry using the water extraction method. As the total sugar concentration of GBCP was relatively high, it was further purified using ethanol treatment, washing, and successive desalting, to produce the GBPP. The yield of GBPP was 11.2% w/w from dried raw ginseng berry. As shown in **Table 1**, The GBPP was composed of 72.8% neutral sugars, 15.2% uronic acids, 7.1% phenolic compounds, and 5.1% protein. Moreover, the GBPP comprised seven kinds of monosaccharides, including rhamnose (8.4%), arabinose (19.5%), xylose (2.2%), mannose (1.5%), galactose (26.6%), glucose (5.4%), and galacturonic acid (15.2%) (**Table 1**). Furthermore, GBPP showed a strong reaction with β-glycosyl Yariv reagent, suggesting the presence of an arabino-β-3,6-galactan moiety (35%) (**Figure 2**).

**Table 1:** Chemical composition and sugar content of ginseng berry polysaccharide portion isolated from ginseng berry.

	GBPP
Yield (%)	11.2
Chemical composition (%)	
Neutral sugar	72.8 ± 6.8
Uronic acid	15.2 ± 0.4
Protein	5.1 ± 1.2
KDO-like materials	–
Phenolic compound	7.0 ± 0.6
Component sugar (mole%)	
Rhamnose	8.4 ± 0.2
Fucose	–
Arabinose	19.5 ± 0.2
Xylose	2.2 ± 0.0
Mannose	1.5 ± 0.2
Galactose	35.8 ± 0.5
Glucose	5.4 ± 0.1
Galacturonic acid + glucuronic acid	15.2 ± 0.4

### Ginseng Berry Polysaccharide Portion Facilitate Anti-Complementary Activities

**Figure 3:**
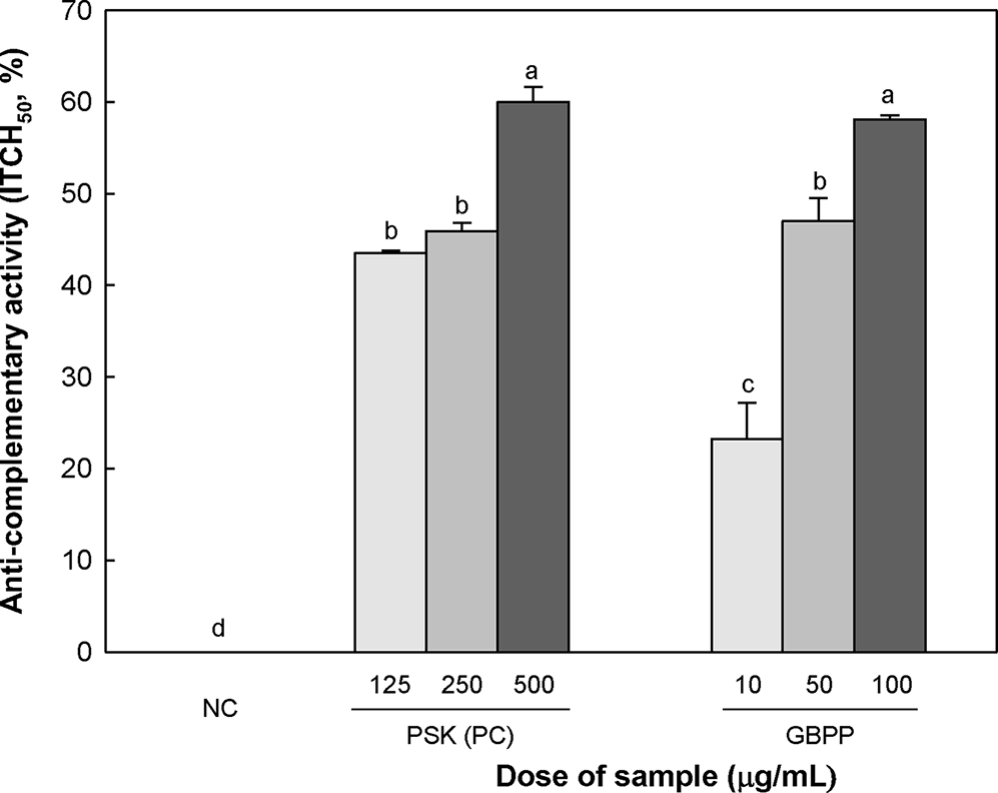
Anti-complementary activity of the ginseng berry polysaccharide portion from ginseng berry. Anti-complementary activity is presented as 50% inhibition of total complementary hemolysis following Mayer´s method. Polysaccharide−K (PSK), a known immunoactive polysaccharide from *Coriolus versicolor*, was used as a positive control and medium was used as a negative control (NC). Values are expressed as a mean ± SD of three independent experiments performed in triplicate. ^a–d^ superscript are significantly different from each other (p < 0.05).

The anti-complementary activities of GBPP are shown in **Figure 3**. The anti-complementary activity was enhanced with increasing GBPP concentration, reaching the maximal activation at 100 µg/ml. At this concentration, GBPP exhibited a higher anti-complementary activity than a similar concentration of polysaccharide-K (PSK), a commercialized immunostimulating polysaccharide from *Coriolus versicolor* (Tsukagoshi and Ohashi, 1974).

### Ginseng Berry Polysaccharide Portion Enhanced Cytokine Production of Macrophage

**Figure 4:**
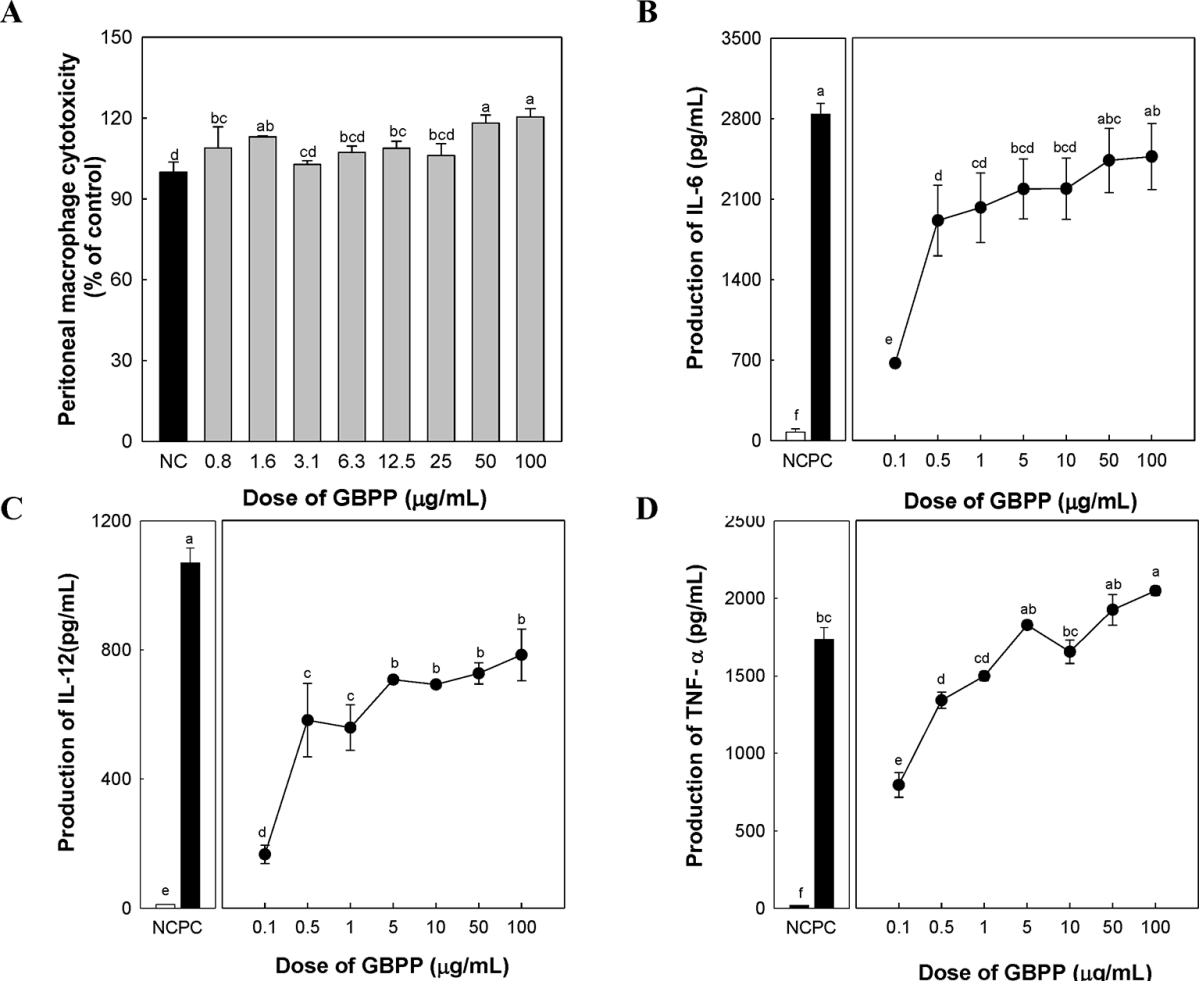
Effect of ginseng berry polysaccharide portion on cytotoxicity **(A)**, and production of cytokines interleukin (IL)-6 **(B)**, IL-12 **(C)**, and tumor necrosis factor-α **(D)** by murine peritoneal macrophages. Values are expressed as a mean ± SD of three independent experiments performed in triplicate. ^a–f^Bars not sharing the same superscript are significantly different from each other (*p* < 0.05).

To investigate the cytotoxicity and cytokine production by GBPP, macrophage proliferation, as well as the levels of three cytokines that are produced by activated peritoneal macrophages, were measured. GBPP showed no cytotoxicity toward macrophages, especially at the higher dose of 100 µg/ml (**Figure 4A**). Compared with a negative control, treatment with GBPP at concentrations of 0.1–100 µg/ml significantly enhanced the cytokine levels of IL-6, IL-12, and TNF-α in a dose-dependent manner (**Figures 4B–D**).

### Effect of Intravenous Ginseng Berry Polysaccharide Portion Administration on Natural Killer Cell Activity

**Figure 5:**
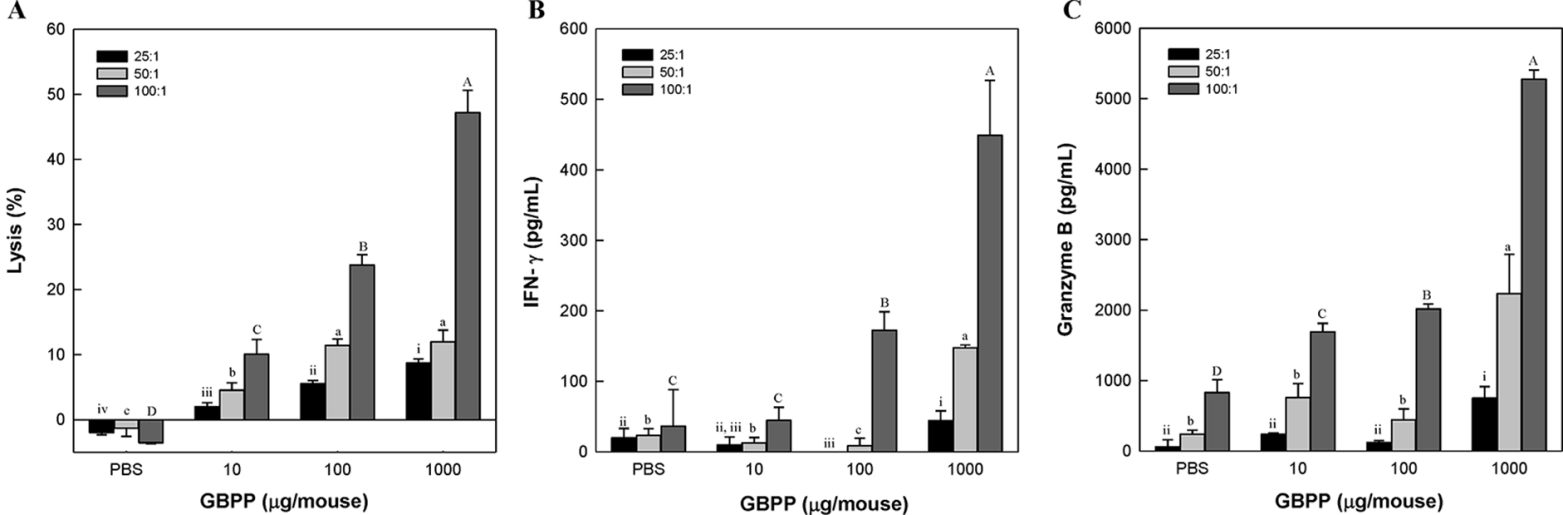
Effects of *i.v.* administration of ginseng berry polysaccharide portion on cytolytic activity **(A)**, and production of IFN-γ **(B)** and granzyme B **(C)** in splenic natural killer cells. Values are expressed as a mean ± SD of three independent experiments performed in triplicate. ^A–D,a–c,i–iv^Bars not sharing the same superscript are significantly different from each other (*p* < 0.05).

NK cells are crucial for immune surveillance against bacteria, tumors, and viral infections (Finlay, 2015; Gardiner and Finlay, 2017). To investigate the activation of NK cells after intravenous administration of GBPP, YAC-1 cells were used. YAC-1 cells are lymphoma cells derived from the Moloney murine leukemia virus, lacking expression of major histocompatibility complex (MHC)-1, which makes them susceptible to NK cell-mediated cell lysis (Kiessling et al., 1975). NK cell-mediated cytotoxicity was investigated in YAC-1 cells co-cultured with NK cells obtained from GBPP-administered mice (*i.v.*), using an LDH release assay. Splenic cells obtained after intravenous GBPP administration to mice at 10, 100, and 1,000 µg/mouse exhibited a stronger cytolytic activity in target cells than in cells obtained from mice treated with PBS in proportion to the E/T ratio (**Figure 5A**). Compared with NK cells from the control mice, cells from GBPP-treated mice showed a significantly higher cytolytic effect in YAC-1 cells. In addition, GBPP enhanced IFN-γ and granzyme B secretion by NK cells, as shown in **Figures 5B, C**. IFN-γ and granzyme B production elevated after 100–1,000 µg/mouse intravenous administration of GBPP at an E/T ratio of 50:1 and 100:1.

### Effect of Ginseng Berry Polysaccharide Portion Administration on Lung Cancer

To investigate the anti-cancer effect of GBPP, B16-BL6 melanoma cells were used as a representative lung cancer cell line. The *p.o.* as well as *i.v.* GBPP-treated mice showed a significant decrease in lung cancer. GBPP delivered by *i.v.* suppressed cancer by approximately 48% at 100 µg/mouse, compared with the control mice (**Table 2**).

**Table 2:** Effect of natural cell deficient on ginseng berry polysaccharide portion-induced inhibition of lung cancer.

Group	Number of lung tumor		
	GBPP (100 μg/mouse)		
	Colony (mean ± SD)	Range (n = 5)	Growth %
Tumor control (untreated)	58.2 ± 6.9	50, 52, 61, 62, 66	100.0 ± 11.8^c^
GBPP only	30.4 ± 4.0	25, 28, 31, 33, 35	50.8 ± 9.4^d^
Anti-asialo GM1 control	131.0 ± 9.2	118, 126, 134, 135, 142	225.1 ± 15.8^a^
Anti-asialo GM1 with GBPP	82.6 ± 6.0	75, 79, 82, 87, 90	141.9 ± 10.4^b^

To deplete NK cells in vivo, rabbit anti-asialo GM1 serum was injected into mice 1 and 3 days before inoculation of B16BL6 melanoma cells. Mice were treated with GBPP (100 μg, i.v.) 2 days before tumor inoculation. Mice were sacrificed 14 days after tumor inoculation for evaluation. ^a∼d^Superscripts express significant differences between groups (P < 0.05), as determined by Duncan’s multiple range test.

To determine whether the anti-cancer effect of GBPP was from NK cell activation, the rabbit anti-asialo GM1 antibody was utilized to block the NK cell activity (Suttles et al., 1986). The anti-cancer effects were then tested using B16-BL6 melanoma cells. The number of tumor colonies in the tumor control group was 58, whereas the number of tumor colonies in the NK cell blocking group treated with anti-asialo GM1 was 131, reflecting a 2.3-fold difference (**Table 2**). Therefore, NK cells play an essential role in the inhibition of tumorigenesis. The number of tumor colonies in the 100 µg/mouse GBPP-treated group was 30. Furthermore, the number of tumor colonies in the GBPP-treated group of NK cell-blocked mice was 82, reflecting a 37% decrease compared with the untreated control group.

**Figure 6:**
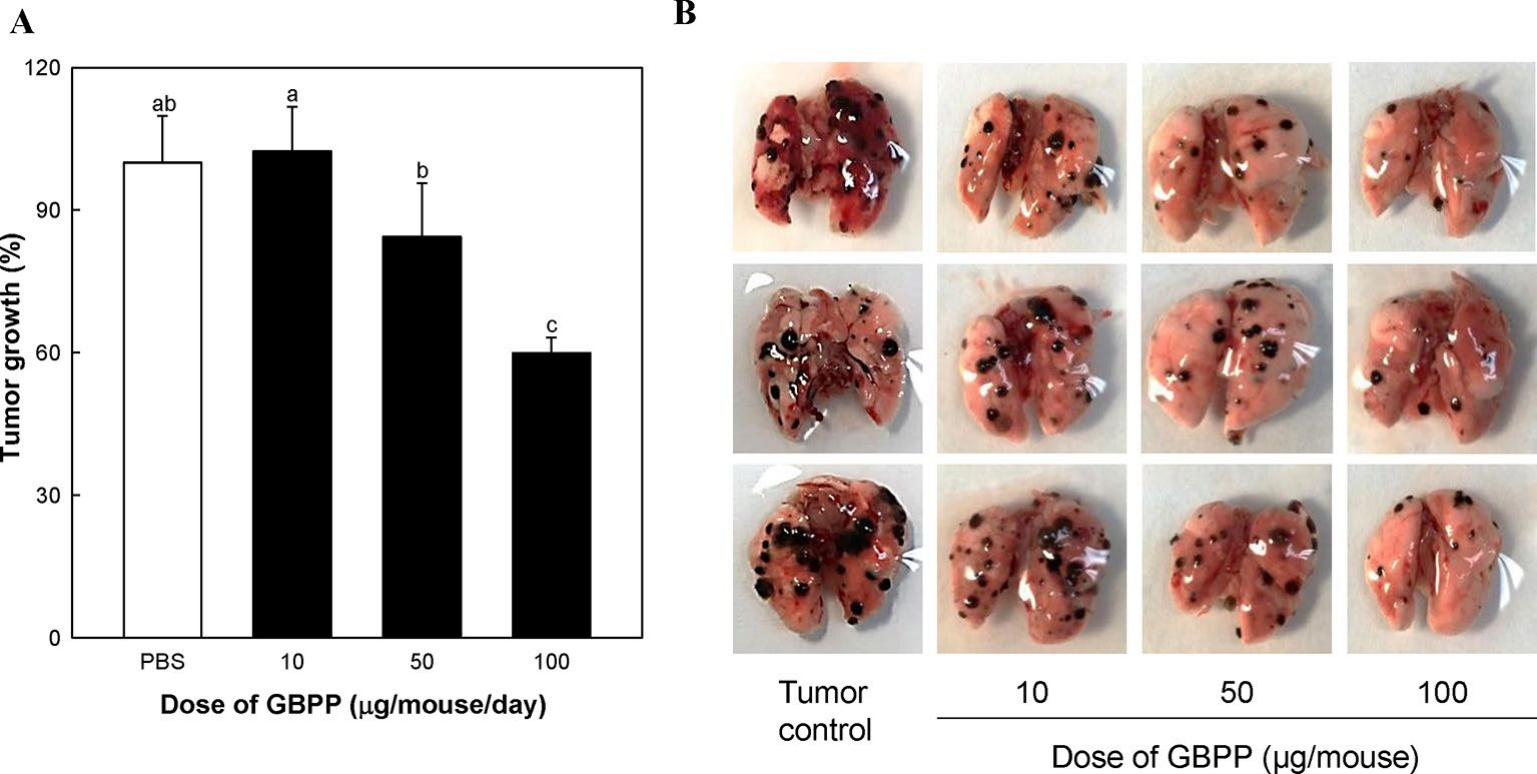
The inhibitory effect of ginseng berry polysaccharide portion on lung cancer produced by inoculation with B16-BL6 melanoma cells. **(A)** Growth rate (%) of lung cancer colonies compared to tumor control (100%). **(B)** Lung photograph excised in each groups. Means with different superscript letters (a–c) indicate significant differences at p < 0.05 by Duncan’s multiple range tests.

We also examined the effects of switching from *i.v.* injection to oral administration of GBPP on lung cancer (**Figure 6**). GBPP treatment prior to B16-BL6 melanoma implantation significantly suppressed the growth of tumor colonies in mice at a concentration of 50 to 100 µg/mouse (P < 0.05). Specially, GBPP 100 µg/mouse treatment group showed an approximate 40% reduction in tumor colonies compared with the untreated group (P < 0.05, **Figure 6A**).

**Figure 7:**
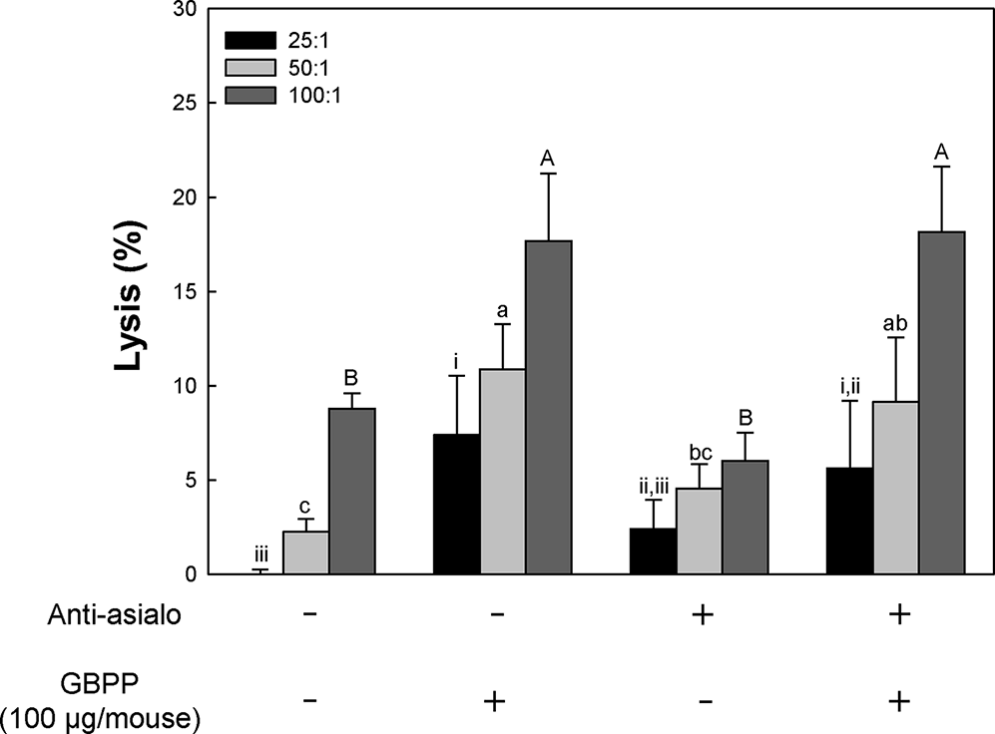
Effect of intravenous administration of ginseng berry polysaccharide portion on cytotoxic T lymphocyte (CTL) activation of normal and natural killer cell depleted BALB/c mice. To deplete NK cells *in vivo*, mouse anti-asialo GM1 serum was injected into mice 2 days before inoculation of B16-BL6 melanoma cells. Mice were treated with sample (100 μg, intravenous) 1 and 3 days before tumor inoculation. And then, splenocytes were harvested and incubation with B16-BL6 cells. Means with different superscript letters (i–iii, a–c, A–B) indicate significant differences at *p* < 0.05 by Duncan’s multiple range test.

Based on above results, GBPP were identified to have anti-cancer effects, associated with the activation of NK cells and other immune cells such as CTL and macrophages. Thus, we aimed to evaluate whether GBPP facilitates CTL activity. As shown in **Figure 7**, although function of NK cell was blocked, GBPP showed higher CTL activity than the untreated control group.

Based on the above CTL activity, GBPP were understood to increase CTL activity in adaptive immunity. This result indicated that adaptive immunity can be increased, together with congenital immune cells, macrophages, and NK cells, to enhance immunity of the host.

## Discussion

Pectic polysaccharides, which are soluble in water, comprise homogalacturonan (HG), rhamnogalacturonan (RG)-I, II domains covalently bound to one another (Voragen, 2009). Previous studies have reported various pharmacological activities of Pectin polysaccharides purified from plants, which are associated with the RG-1 and RG-II domains rather than the HG domain (Srivastava and Kulshreshtha, 1989).

In this study, as GBPP included a high arabinose content and galactose, we performed single radial gel diffusion test with β-glucosyl Yariv reagent for the identification of arabinogalactan (AG)-II. The Yariv reagent has the ability to precipitate polysaccharides containing AG-II (Holst and Clarke, 1985; Braünlich et al., 2018). As shown in **Figure 2**, a positive correlation was observed between the Yariv reagents in the standard reference gum arabic and the area of precipitation measured as the squared diameter of the corona shaped in the radial diffusion test. RG-I is composed of [→2)-α-L-Rhap-(1 → 4)-α-D-GalpA-(1→] repeating units as backbone, decorated with Ara- and Gal-containing side chains at O-4 of Rha residues. The side chains of RG-I consist of arabinogalactan I or II (AG-I or AG-II), arabinan, and galactan (Mohnen, 2008). The GBPP exhibited a high content of AG-II (35%), suggesting that the AG-II moiety is important for expressing the immunostimulatory activity in GBPP. Furthermore, GBPP may contain mainly pectic components, the complicated and glycosidic macromolecules discovered in the unlignified cell wall of plants (Voragen, 2009).

The complement system was originally revealed as a heat-sensitive element in fresh serum that “complemented” the effects of particular antibody during the lysis of red blood cells and bacteria (West et al., 2018). It is an important effector of the humoral immunity (Noris and Remuzzi, 2013) and represents a highly integrative system with a number of functions, including removal of injured cells and debris, host defense, regenerative processes, modulation of metabolism, and regulation of adaptive immunity (Reichhardt and Meri, 2018). Ginsenan, acidic polysaccharides from ginseng root, was reported to enhance reticuloendothelial activity and promote anti-complement activities (Tomoda et al., 1993a; Tomoda et al., 1993b). In this study, GBPP showed a strong anti-complementary effect compared with a dosage of PSK. In addition, GBPP showed an anti-complement effect at 10% of concentration compared to the ginsenan. Many studies have already reported the anti-complementary effect of polysaccharides isolated from natural products (Lee et al., 2018). Lee et al. reported that the four ginsenosides, Rg6, F4, Rk3, and Rh4, have an inhibitory effect on complement activation through classical pathways (Lee et al., 2011).

Macrophages play a critical role in many diseases, and have therefore, emerged as attractive targets for therapy. Monocytes circulate and migrate into tissues during infection and inflammation, where they differentiate into macrophages (Layoun et al., 2015). Activated macrophages, when stimulated, produce IL-6, IL-12, TNF-α, granulocyte colony-stimulating factor, and gamma interferon (Voskoboinik et al., 2015). As a polysaccharide of ginseng root, it has been studied that ginsan induces expression of TNF-α, IFN-γ, and inducible nitric oxide synthase mRNAs in splenocytes and peritoneal macrophages of mice (Lee et al., 1997). In the present study, the GBPP was found to induce a potent host-protective immune response by up-regulating pro-inflammatory cytokines involving IL-6, IL-12, and TNF-α in murine peritoneal macrophages. Furthermore, the production of IL-6 and IL-12 were lower than the levels generated by 1 µg/ml LPS, indicating that inflammation pathways were not included in this response. IL-6 is also regarded as an important immune and inflammatory factor (Tanigawa et al., 2000).

NK cells are capable of elicit the lysis and death of target cells by producing granzyme and perforins (Voskoboinik et al., 2015). Moreover, NK cells present anti-cancer and anti-viral effect by releasing various pro-inflammatory factors involving IFN-γ (Wang et al., 2018; Zhang et al., 2018). Previous studies have shown that ginseng and its major active components are responsible for the chemo-preventive effects following the activated NK cells (Choi et al., 2017; Shin et al., 2017). The present results also proved that GBPP promotes NK cell cytotoxicity *via* the release of IFN-γ and granzyme B. These findings suggest that GBPP treatment could involve harnessing this NK cell-related immune regulation. Dendritic cells or macrophages, including cancer cells, form the first defense against external invasion and are a major component of the innate immune system (Clark et al., 2000). These phagocytes use pattern recognition receptors such as toll-like-receptors to recognize immunosimulants and simultaneously produce immune mediators such as cytokines. Additionally, cytokines, involving interleukin (IL)-12, can activate natural killer (NK)-cells, which play an important role in cytotoxicity against cancer cells (Lee et al., 2014). Some recent studies have revealed macrophage polarization, where macrophages differentiate into tumor-associated macrophages (TAM) in the presence of cancer cells (Solinas et al., 2010). Certain activation signals may induce polarized activation macrophages. The typically activated macrophages (M1) have an increased expression of major histocompatibility (MHC) class II, IL-12 and TNF-α, production of reactive oxygen, and nitric oxide (Liu et al., 2015). It seems that the anti-cancer actions of the administered polysaccharide, GBPP, isolated from ginseng berry be due to the enhancement of the activity of innate immune cells, such as macrophages and NK-cells. In addition, GBPP is believed to be involved in the polarization of macrophages to the M1 type by promoting the production of cytokines, such as TNF-α and IL-12. Also, when macrophages recognize non-self cells and phagocytes, they kill the phagocytic cells using proteolytic enzymes. At the same time, peptides degraded from non-self cells is presented to MHC class I and II for generating the adaptive immune response (Iwasaki and Medzhitov, 2010). Thereafter, antigen-recognized native T lymphocytes are differentiated into cytotoxic T lymphocytes (CTL), helper T lymphocytes, and memory T lymphocytes, eventually leading to adaptive immunity (Hoebe et al., 2004).

Previous studies have also demonstrated anti-cancer and anti-metastatic effects of ginseng, as well as ginseng byproducts and their bioactive components (Wong et al., 2015; Cho et al., 2017; Lee et al., 2017). Song et al. investigated that ginsan enhanced the peritoneal macrophage secretory and tumoricidal activities (Song et al, 2002). In addition, ginsan was reported to show significant *in vivo* antitumor activities against B1 melanoma cells and in the benzo(a)pyrene-induced lung tumor model (Lee et al., 1997). Recently, biologically active polysaccharides have received great attention in a variety of remedies for cancer-related diseases (Liu et al., 2018). Furthermore, bioadhesive polysaccharides have generated considerable interest as auxiliary agents for oral administration (Agrawal et al., 2017). Taking together our results on immunostimulant and anti-cancer effects, GBPP may have broad application perspectives in the treatment of cancer and immunodeficiency diseases.

## Conclusion

The present study demonstrated that GBPP tended to increase the anti-complementary activity and peritoneal macrophage activation. In addition, the results also showed that GBPP promotes murine peritoneal macrophage activation and NK cell cytotoxicity. Furthermore, GBPP *i.v.* and *p*.o. administration prior to B16-BL6 melanoma implantation significantly suppressed the growth of tumor colonies in mice. These results suggest the potential for GBPP as a therapeutic agent for cancer prevention and inhibition, *via* regulation of immune responses.

## Data Availability Statement

The datasets supporting the conclusions of this article are included within the article.

## Ethics Statement

All the experiments were approved by the Kyonggi University Institutional Animal Care and Use Committee (2016-002 & 2017-005).

## Author Contributions

K-SS and JP conceived and designed the experiments. D-YL, SL, H-RP, and S-US conducted the experiments, analyzed the data, and generated the figures and tables. K-SS and JP supervised the whole project, and CP and SK wrote the manuscript. All authors read and approved the final manuscript.

## Conflict of Interest

Authors CP and SK were employed by Amore Pacific Corp.

The authors declare that the research was conducted in the absence of any commercial or financial relationships that could be construed as a potential conflict of interest.

## Abbreviations

AG, arabinogalactan; CCK-8, cell counting kit-8; ELISA, enzyme-linked immunosorbent assay; FBS, fetal bovine serum; FID, flame ionization detector; GBPP, ginseng berry polysaccharide portion; GC, gas chromatography; GVB^2+^, gelatin-veronal buffered saline; HG, homogalacturonan; IFN, interferon; IL, interleukin; ITCH_50_, inhibition of 50% of total complement hemolysis; KDO, 2-*keto*-3-deoxy-D-manno-octulosonic acid; LDH, lactate dehydrogenase; LPS, lipopolysaccharide; MW, molecular weight; NHS, normal human serum; NK, natural killer; PSK, polysaccharide-K; RG, rhamnogalacturonan; RPMI, Roswell Park Memorial Institute; TBA, thiobarbituric acid; TNF, tumor necrosis factor.
